# A hybrid exercise-based cardiac rehabilitation program is an effective strategy to improve muscle strength and functional exercise capacity in adults and older people with coronary artery disease

**DOI:** 10.3389/fphys.2022.948273

**Published:** 2022-08-05

**Authors:** Gabriel Nasri Marzuca-Nassr, Pamela Seron, Claudia Román, Manuel Gálvez, Rocío Navarro, Gonzalo Latin, Tania Marileo, Juan Pablo Molina, Pablo Sepúlveda, María José Oliveros

**Affiliations:** ^1^ Departamento de Ciencias de la Rehabilitación, Facultad de Medicina, Universidad de La Frontera, Temuco, Chile; ^2^ Departamento Ciencias de la Salud, Facultad de Medicina, Pontificia Universidad Católica, Santiago, Chile; ^3^ Complejo Hospitalario San José, Santiago, Chile; ^4^ Hospital Clínico Universidad de Chile, Santiago, Chile; ^5^ Hospital Clínico San Borja-Arriarán, Santiago, Chile; ^6^ Hospital Regional de Antofagasta, Antofagasta, Chile; ^7^ Hospital San Juan de Dios, Santiago, Chile

**Keywords:** aging, elderly, coronary hearth disease, exercise, physical performance

## Abstract

Coronary heart disease is the most common cause of death worldwide. Standard cardiac rehabilitation (face-to-face sessions) has shown benefits in increasing muscle strength and functional exercise capacity in adults and older people. However, it is unknown whether hybrid cardiac rehabilitation (a first face-to-face phase + a second remote monitoring phase) will have similar benefits in adults versus older subjects. The aim of this study was to compare the effects of a hybrid exercise-based cardiac rehabilitation program on muscle strength and functional exercise capacity in “adult” versus “older” people with coronary artery disease. We hypothesized that a hybrid exercise-based cardiac rehabilitation program would improve muscle strength and functional exercise capacity, but the impact would be smaller in the older group than the adult individuals. This study is part of a larger project (The Hybrid Cardiac Rehabilitation Trial-HYCARET). We subjected 22 adult (<60 y) females and males (ADULT; *n* = 5/17 (f/m); 52 ± 5 y; 28.9 ± 3.4 kg·m-2) and 20 older (≥60 y) females and males (OLDER; *n* = 6/14 (f/m); 66 ± 4 y; 27.4 ± 3.9 kg·m-2) with coronary artery disease to 12 weeks of hybrid exercise-based cardiac rehabilitation program. Prior to and after 12 weeks of a hybrid exercise-based cardiac rehabilitation program, grip strength (handgrip), leg strength (chair stand test), and functional exercise capacity (6-minute walk test, 6MWT) were assessed. The hybrid exercise-based cardiac rehabilitation program resulted in a 9.4 ± 14.6% and a 6.2 ± 12.1% grip strength increase, a 14.4 ± 39.4% and a 28.9 ± 48.1% legs strength increase, and a 14.6 ± 26.4% and a 6.8 ± 14.0% functional exercise capacity improvement in ADULT and OLDER, respectively (*p* < 0.05) with no differences between groups. In conclusion, a hybrid exercise-based cardiac rehabilitation program could increase muscle strength and improve functional exercise capacity in adults and older people with coronary artery disease. More future studies comparing effectiveness among these age groups are needed to strengthen this conclusion.

## Introduction

Coronary heart disease is the most common cause of death worldwide (Dibben et al., 2021). In this sense, cardiovascular diseases represent 32% of global deaths of people in productive age and an estimated 17.9 million people died from these condition in 2019 (WHO, 2021). In Chile, cardiovascular diseases are the main cause of death, representing 27.1% of all deaths in 2016 (Troncoso-Pantoja et al., 2020). With advances in medical treatment, patients have better access to acute revascularization treatments such as thrombolysis and angioplasty, and therefore achieve better outcomes and survival. For example, in Brazil, the crude mortality rates from coronary heart disease decreased in both sexes and in all age groups (only an increase of 1.78% was observed in males over 85 y) (Moreira et al., 2021). Similarly, Argentina and Colombia showed declines of 51% and 6.5%, respectively. However, despite this declining trend, interventions such as cardiac rehabilitation have been shown to be effective reducing cardiovascular mortality, even in the angioplasty era (RR = 0.48; 95% CI 0.28–0.83) (Oliveros et al., 2022a). On the contrary, in some countries such as Mexico the mortality rate increases in 61% (Arroyo-Quiroz et al., 2020). The above still means a greater number of people must live with the signs and symptoms of coronary disease (Dibben et al., 2021).

Among the treatment strategies, cardiac rehabilitation based on physical exercise has been shown to improve muscle strength (Pratesi et al., 2019), increase functional exercise capacity (Baldasseroni et al., 2016; Pratesi et al., 2019), and improve quality of life (Dibben et al., 2021) in adults and older people. Cardiac rehabilitation (associated with an improvement in muscle strength and function exercise capacity) reduces mortality by 34% versus 21% in patients who do not undergo cardiac rehabilitation. These results are similar to those observed in younger patients (Suaya et al., 2009).

Alternatives to standard rehabilitation (face-to-face center-based sessions) have been proposed, such as telerehabilitation, messaging through the use of mobile devices, among others (Hwang et al., 2017; Pratesi et al., 2019; Seron et al., 2021). A recent rapid overview from our research group concluded that telerehabilitation for cardiac rehabilitation could be comparable to performing face-to-face cardiac rehabilitation or better than not performing cardiac rehabilitation (Seron et al., 2021).

Combining a first phase of rehabilitation where face-to-face sessions and a second phase with remote accompaniment are carried out can be called “hybrid rehabilitation”. This hybrid cardiac rehabilitation yields similar benefits to the training program in the center for patients with coronary disease (Pratesi et al., 2019), reducing costs and human resources and freeing up time for patients.

On the other hand, it is international knowledge that our population is aging and a large percentage of older people have or will have coronary heart disease (Khan et al., 2020). Therefore, actions must be taken to promote healthy aging as a process of maintaining functional capacity to enable well-being in old age (Rudnicka et al., 2020). In line, our main focus is to try to increase the adherence of the older people to cardiac rehabilitation and we believe that using a hybrid program can benefit this population. The beneficial results of this study will be helpful to develop individualized protocol for population with different age. There are reports showing that standard cardiac rehabilitation is similarly beneficial to a lesser (Vilela et al., 2020) or greater (Lavie and Milani, 2000) extent in older people when compared to young people or adult. In addition, recent studies have shown a similar benefit between performing the cardiac rehabilitation program in the center vs. as a hybrid program (Pratesi et al., 2019) or by telerehabilitation (Kraal et al., 2014; Hwang et al., 2017) including people over 60 years. However, to date it is unknown whether hybrid cardiac rehabilitation will have similar benefits in older people (≥60 y) compared to adults with coronary artery disease.

Therefore, in the present study we aimed to compare the effects of a hybrid exercise-based cardiac rehabilitation program on muscle strength and functional exercise capacity in “adult” versus “older” people with coronary artery disease. Secondarily, we also looked at blood pressure and body composition results. We hypothesized that a hybrid exercise-based cardiac rehabilitation program would improve muscle strength and functional exercise capacity, but the impact would be smaller in the older group than the adult individuals.

## Materials and methods

### Participants

Forty-two participants, 22 adult (<60 y) females and males (ADULT; *n* = 5/17 (f/m); 52 ± 5 y; 28.9 ± 3.4 kg·m-2) and 20 elderly (≥60 y) females and males (OLDER; *n* = 6/14 (f/m); 66 ± 4 y; 27.4 ± 3.9 kg·m-2) with coronary artery disease were included. This study is part of a larger project (The Hybrid Cardiac Rehabilitation Trial-HYCARET) that already has prior publications (Seron et al., 2019; Oliveros et al., 2022b). The study was performed in accordance with the Declaration of Helsinki and was approved by the Scientific Ethics Committee (SEC) of the Universidad de La Frontera, Temuco, Chile (registration number Record NoX032-18, Page No016_18); Hospital San Borja Arriarán: SEC of the Central Metropolitan Health Service registration number Record No92/6, Page No618/2018); Hospital San José: SEC of the North Metropolitan Health Service registration number Record No056/2018); Hospital Clínico Universidad de Chile: SEC of Hospital Clínico Universidad de Chile registration number Record No47); and SEC of Hospital Hernán Henríquez Aravena registration number Record No0267). Additionally, the trial was registered on clinicaltrials.gov as NCT03881150. All volunteers performed 20 sessions of hybrid exercise-based cardiac rehabilitation; 10 sessions on-site plus 6 weeks of distance monitoring through text messages and phone calls. For this study, before and after 12-week measurements were analyzed.

### Screening

Prior to the study, volunteers’ suitability to participate was assessed in a single screening session. After explaining all procedures, written informed consent was obtained from subjects willing to participate. The inclusion criteria were: >18 y; patient with coronary artery disease, including acute coronary syndrome (unstable angina, myocardial infarction with or without ST elevation) or stable coronary disease diagnosed by angiography or a stress test; patient treated medically (i.e., medication only) or by thrombolysis, angioplasty or revascularization surgery; patient with physician referral, who can start cardiac rehabilitation between 2 weeks and 2 months from their event, diagnosis or procedure; patient able to attend the health center almost twice a week over 4–6 weeks; and patient with a mobile phone. Exclusion criteria were: patient has a planned repeat cardiac or other procedure in next 12 months; explicit contraindication to perform exercise based on American College of Sport Medicine guidelines (Riebe et al., 2015); patient with comorbidities that would interfere with ability to engage in cardiac rehabilitation such as dementia, blindness, deafness, serious mental illness, or frailty; and musculoskeletal disease that precludes the patient from performing exercise.

### Hybrid exercise-based cardiac rehabilitation program

Ten supervised sessions by a physiotherapist were carried out in a period of 4–6 weeks. Each session included aerobic and resistance training. The training intensity was moderate, starting the rehabilitation program with a duration of 10 min per session up to 60 min per session according to tolerance. In addition to the above, throughout the exercise sessions, self-efficacy-based counseling was provided by the physiotherapist on physical activity, diet, tobacco and medication use.

After 6 weeks of face-to-face rehabilitation, participants were encouraged to maintain the same exercise prescription, eat healthy and have good adherence to their prescribed medications through phone calls twice a week and text messages three times a week for 6 weeks (Seron et al., 2019).

### Strength assessment

Upper and lower body strength were assessed through grip strength and the chair stand test, respectively. Grip strength was performed with a Jamar® Plus+ electronic handheld dynamometer (Patterson Medical, Cedarburg, WI, United States) with the participant seated. Three attempts were made on each hand alternately with 30 s of rest. The highest value of the 6 attempts was reported (Roberts et al., 2011). The chair stand test was performed (also used by some authors as an indicator of physical performance or power) with the participant in a seated position in a chair without armrests and without wheels, feet flat on the floor and arms crossed on the chest. From this position the participant had to rise fully and return to the starting position as many times as possible during 30 s. The repetitions achieved are reported (Rikli and Jones, 2013).

### Functional exercise capacity

The 6-minute walking test (6MWT) was conducted following the recommendations of the ATS Statement (Laboratories, 2002). In short, in a 30-meter straight corridor, the participant walked the greatest possible distance during the 6-minute duration of the test. At the end of the test, the participant was notified and the place where they stopped was marked to measure the distance covered in meters.

### Blood pressure and body composition

Body weight, height, blood pressure, and waist circumference were assessed by a trained evaluator. Body mass index (BMI) was determined by weight in kilograms divided by height in meters squared. Body weight measurement was performed with the least possible clothing. Height was assessed with the participant barefoot, looking straight ahead at a fixed point, and in inspiration. Both measurements were made on a balance with a stadiometer (SECA®, Madison, WI, United States). Measurement of blood pressure was performed through an automatic blood pressure monitor (Omron, HEM-7120, Japan) on the participant’s right arm, after remaining seated for 5 min (Pickering et al., 2005). Waist circumference was assessed directly on the participant’s body. The participant was asked to cross their arms to their chest, touching their shoulders. The measurement was made at the midpoint of the right side of the body, between the last rib and the upper border of the iliac crest. The participant had to exhale (Seron et al., 2019).

### Analysis

Data are presented as mean ± standard deviation. To analyze the normality of the data, the Shapiro-Wilk test was used. At baseline, data between groups were compared with an independent samples t-test for quantitative variables and a Chi-square test for qualitative variables. Pre- versus post-intervention data were analyzed using a repeated-measures ANOVA with time (PRE versus POST) as the within-subjects factor and group (ADULT versus OLDER) as the between-subjects factor. In the case of a significant interaction, paired t-tests were performed to determine time effects within groups and independent t-tests for group differences in the PRE, and POST-evaluations values, using an α level of 0.05. Additionally, a partial eta squared (η2) was used to estimate effect sizes for ANOVA and Cohen’s d (d) for t-test. All calculations were performed using SPSS version 24.0 (IBM Corp., Armonk, NY, United States).

## Results

### Participants

**FIGURE 1:**
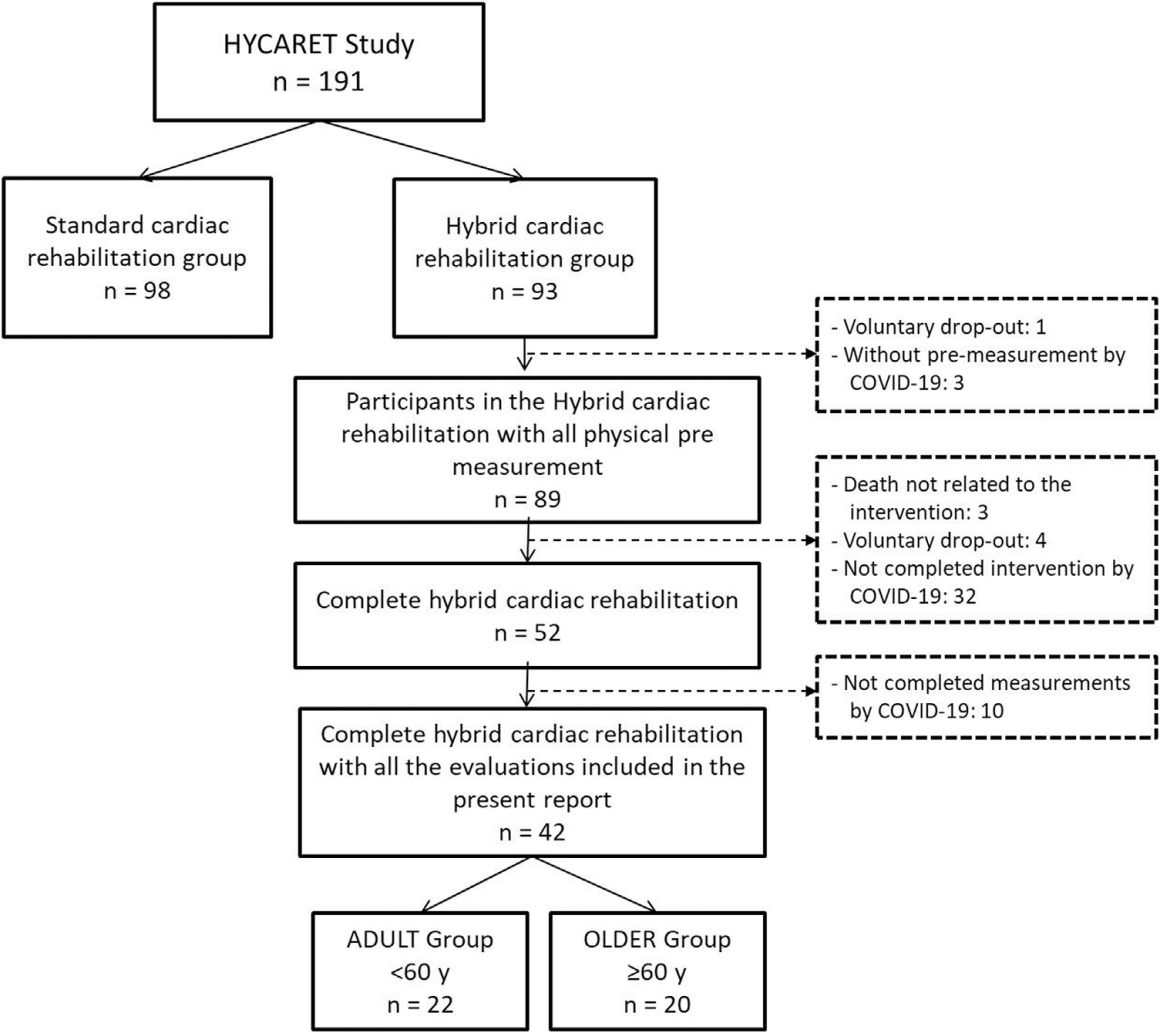
Flow diagram of the study participants.

In the HYCARET study (standard vs. hybrid cardiac rehabilitation), 191 underwent a cardiac rehabilitation program. A subsample of 42 participants, 22 participants in the ADULT group (<60 years) and 20 participants in the OLDER group (≥60 years) in the hybrid cardiac rehabilitation group had completed all the evaluations included in the present report. The flow diagram of the study participants is shown in Figure 1. Participants’ characteristics are shown in Table 1, observing a significant difference only for age (*p* < 0.001).

**TABLE 1:** Participants’ characteristics.

	ADULT (*n* = 22)	OLDER (*n* = 20)	*p*
Age (y)	52 ± 5	66 ± 4	0.000
Weight (kg)	77.3 ± 12.5	72.8 ± 12.7	0.246
Height (cm)	163.6 ± 7.8	162.8 ± 9.2	0.752
BMI (kg.m-2)	28.9 ± 3.4	27.4 ± 3.9	0.203
SBP (mmHg)	121.5 ± 20.6	126.8 ± 19.1	0.390
DBP (mmHg)	76.6 ± 12.1	73.0 ± 9.4	0.287
Waist circumference (cm)	98.0 ± 9.4	97.7 ± 9.6	0.919
Men	17 (77.3%)	14 (70.0%)	0.592
Comorbidities			
AH	15 (68.2%)	14 (70.0%)	0.920
DM	3 (13.6%)	6 (30.0%)	0.197
DLP	6 (27.3%)	8 (40.0%)	0.342
COPD	1 (4.5%)	2 (10.0%)	0.137
CVA	0	1 (5.0%)	0.288
Asthma	1 (4.5%)	1 (5.0%)	0.915
AMI previous	8 (36.4%)	3 (15.0%)	0.165
CA	1 (4.5%)	1 (5.0%)	0.300

Data are means ± SD, and frequency (percentage). Data were analyzed using an independent samples t-test for quantitative variables and a Chi-square test for qualitative variables.

BMI, body mass index; SBP, systolic blood pressure; DBP, diastolic blood pressure; y, years; cm, centimeters; kg, kilograms; m, meters; mmHg, millimeters of mercury; AH, arterial hypertension; DM, diabetes mellitus; DLP, dyslipidemia; COPD, chronic obstructive pulmonary disease; CVA, cerebrovascular accident; AMI, acute myocardial infarction; CA, cancer.

### Strength

**FIGURE 2:**
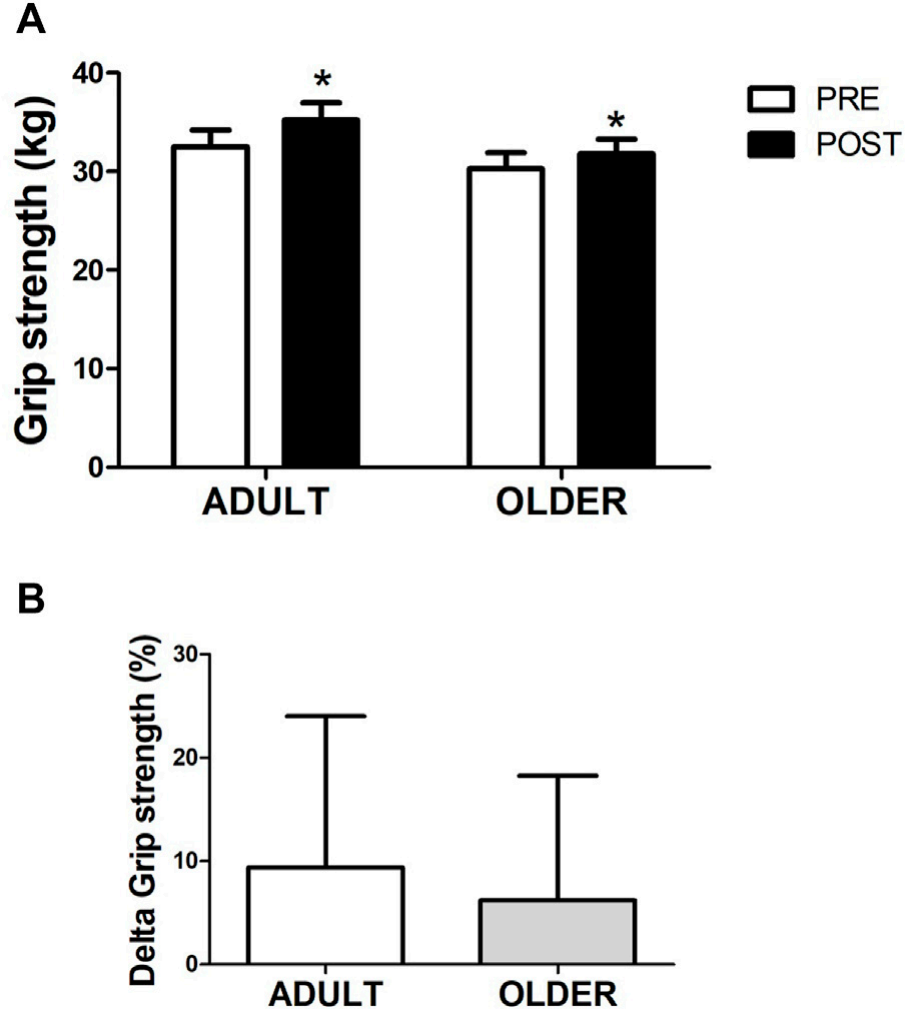
Strength through maximal handgrip strength **(A)**. Percentage of variation between PRE and POST in grip strength **(B)** is also shown. *n* = 22 participants in the ADULT group and *n* = 20 participants in the OLDER group. Data were analyzed using a repeated-measures ANOVA **(A)** and an independent samples t-test **(B)**. * *p* < 0.05 (time effect).

**FIGURE 3:**
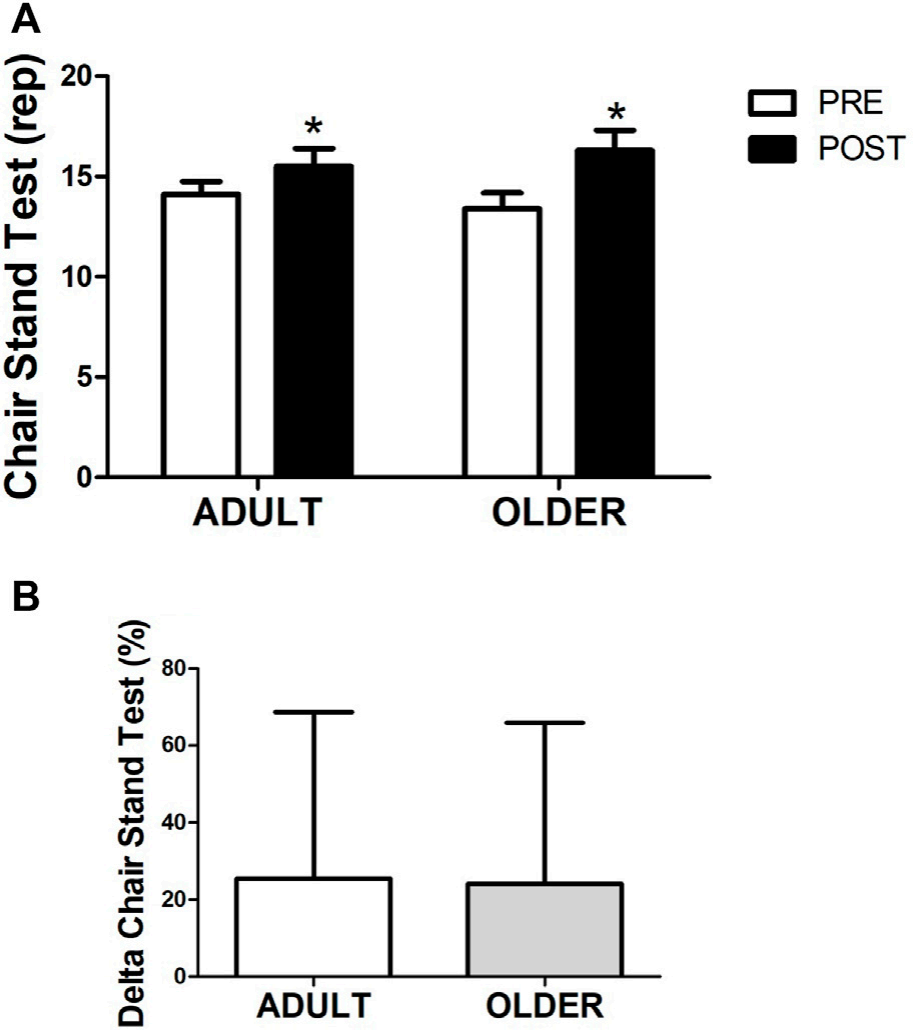
Strength through chair stand test **(A)**. Percentage of variation between PRE and POST in leg strength **(B)** is also shown. *n* = 20 participants in the ADULT group and *n* = 19 participants in the OLDER group. Data were analyzed using a repeated-measures ANOVA **(A)** and an independent samples t-test **(B)**. * *p* < 0.05 (time effect).

After the hybrid exercise-based cardiac rehabilitation program, grip strength (Figure 2) increased from 32.5 ± 8.1 to 35.2 ± 8.3 kg (9.4 ± 14.6%) in the ADULT group and from 30.3 ± 7.2 to 31.8 ± 6.7 kg (6.2 ± 12.1%) in the OLDER group (time effect, *p* = 0.001; η2 = 0.26). Similarly, 12 weeks of cardiac rehabilitation program resulted in an increase in legs strength (Figure 3) from 14.1 ± 2.9 to 15.5 ± 4.0 rep (14.4 ± 39.4%) in the ADULT and from 13.4 ± 3.4 to 16.3 ± 4.4 rep (28.9 ± 48.1%) in the OLDER (time effect, *p* = 0.014; η2 = 0.15). No differences in the response to the cardiac rehabilitation program were observed between groups in all strength variables (time∗group interaction effect, all *p* > 0.05; all η2≤0.03).

Accordingly, the delta increase in grip and legs strength did not differ between ADULT and OLDER (*p* > 0.05) and the effect sizes were d = 0.24 and d = 0.33, respectively.

### Functional exercise capacity

**FIGURE 4:**
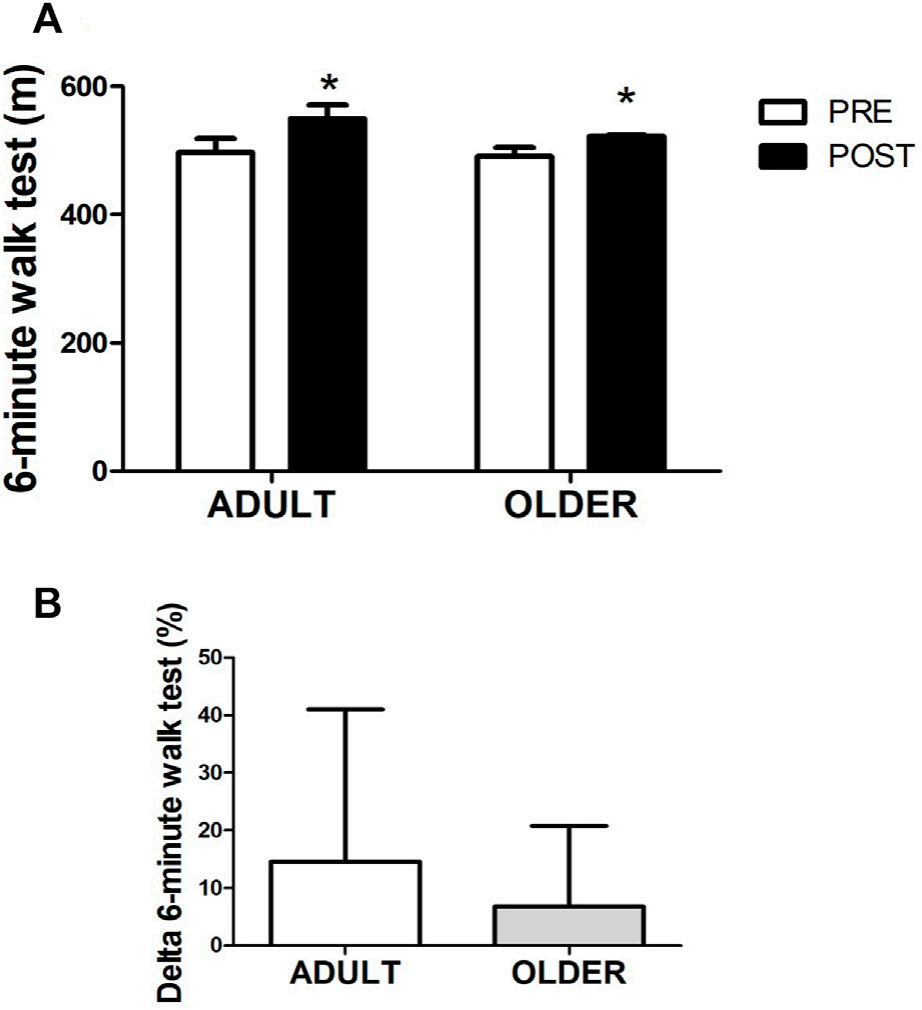
Functional exercise capacity through 6-minute walk test **(A)**. Percentage of variation between PRE and POST in exercise capacity **(B)** is also shown. *n* = 20 participants in the ADULT group and *n* = 19 participants in the OLDER group. Data were analyzed using a repeated-measures ANOVA **(A)** and an independent samples t-test **(B)**. * *p* < 0.05 (time effect).

Hybrid exercise-based cardiac rehabilitation program increased functional exercise capacity after 12 weeks (Figure 4), from 496.8 ± 98.5 to 549.8 ± 96.3 m in the ADULT and from 490.7 ± 60.5 to 522.3 ± 84.8 m in the OLDER (time effect, *p* = 0.009; η2 = 0.18) with no differences between groups (time*group interaction effect, *p* = 0.489; η2 = 0.01). Accordingly, the relative increase in functional exercise capacity did not differ between ADULT (14.6 ± 26.4%) and OLDER (6.8 ± 14.0%; *p* > 0.05; d = 0.37).

### Blood pressure and body composition

After the hybrid exercise-based cardiac rehabilitation program, no significant differences were observed in weight, BMI, blood pressure (systolic blood pressure, SBP; diastolic blood pressure, DBP), or waist circumference between ADULT vs. OLDER (all *p* > 0.05; all η2≤0.09; Table 2).

**TABLE 2:** Blood pressure and body composition parameters before and after hybrid exercise-based cardiac rehabilitation program.

	ADULT (*n* = 22)			OLDER (*n* = 20)			Statistics		
	PRE	POST	Delta PRE-POST (%)	PRE	POST	Delta PRE-POST (%)	Time	Time × Group	Group
Weight (kg)	77.3 ± 12.5	78.3 ± 13.3	1.2 ± 4.7	72.8 ± 12.7	72.9 ± 13.4	0.2 ± 5.4	0.354	0.499	0.216
BMI (kg.m-2)	28.9 ± 3.4	28.7 ± 3.9	-0.5 ± 5.4	27.4 ± 3.9	27.8 ± 4.3	1.5 ± 6.3	0.584	0.268	0.314
SBP (mmHg)*	121.5 ± 20.6	126.2 ± 16.6	5.9 ± 17.8	128.4 ± 18.1	124.4 ± 18.1	-1.9 ± 16.5	0.919	0.193	0.589
DBP (mmHg)*	76.6 ± 12.1	78.1 ± 9.6	3.6 ± 15.3	74.2 ± 7.7	74.6 ± 12.9	1.0 ± 17.2	0.623	0.782	0.299
Waist circumference (cm)	98.0 ± 9.4	99.3 ± 8.9	1.5 ± 5.1	97.7 ± 9.6	99.3 ± 11.7	1.5 ± 4.6	0.054	0.849	0.958

*Nineteen participants in each group were considered for the analysis.

Data are means ± SD. Data were analyzed using repeated measures ANOVA (time x group).

BMI, body mass index; SBP, systolic blood pressure; DBP, diastolic blood pressure; cm, centimeters; kg, kilograms; m, meters; mmHg, millimeters of mercury.

## Discussion

The primary aim of this study was to compare the effects of a hybrid exercise-based cardiac rehabilitation program on muscle strength and functional exercise capacity in ‘adult’ versus ‘older’ people with coronary artery disease. We hypothesized that 1) hybrid exercise-based cardiac rehabilitation program would improve muscle strength and functional exercise capacity in both groups, and 2) the impact after cardiac rehabilitation program on muscle strength and functional exercise capacity would be smaller in the older group when compared to the adult individuals.

Due to limited time and resources and the health pandemic we are experiencing (COVID-19), new ways of delivering therapeutic strategies in patients with coronary artery disease have emerged. One of the proposed strategies is hybrid cardiac rehabilitation. This study evidences that adult and older patients with coronary artery disease benefit in their gains in muscle strength and functional exercise capacity after 12 weeks of a hybrid exercise-based cardiac rehabilitation program.

The normal aging process leads to a decrease in muscle mass, strength and physical performance (Marzuca-Nassr et al., 2020). In older people with coronary artery disease, there will be a worsening of functional exercise capacity due to decreased outcomes as well as coronary artery disease itself (Lavie and Milani, 2000). Due to the aforementioned, we hypothesized that older people would present a beneficial response, but to a lesser extent when compared to adults due to the normal differences in strength and functional exercise capacity between adult versus older people. We have observed that the exercise response to hybrid cardiac rehabilitation in the elderly is preserved. This opens up a great possibility of strategies, such as the one carried out in this report, to favor the elderly population.

Strategies like exercise-based cardiac hybrid rehabilitation have turned out to be an effective strategy to improve strength and functional exercise capacity in older people. The Cochrane Review update on this topic supports that cardiac rehabilitation benefits people with coronary heart disease, reducing the risk of myocardial infarction, somewhat reducing all-cause mortality, and greatly reducing all-cause hospitalization, thereby reducing health costs and improving quality of life at 12 months (Dibben et al., 2021).

Despite all the benefits reported by cardiac rehabilitation, it is underutilized in older people. For this reason, new delivery strategies like hybrid cardiac rehabilitation could help increase adherence and reach of this type of therapy to the elderly population (Lutz and Forman, 2022). In addition to the above, no differences were observed in the present study in the benefits of hybrid cardiac rehabilitation between adults and older people. Therefore, both benefit after hybrid cardiac rehabilitation. Improving muscle strength and functional exercise capacity will help older people to carry out their activities of daily living independently and thus improve their quality of life.

Hwang et al. (2017) concluded that cardiac rehabilitation through telerehabilitation (home-based rehabilitation program) does not produce benefits inferior to standard cardiac rehabilitation (center-based rehabilitation program) in patients with stable chronic heart failure (67 ± 12 y) (Hwang et al., 2017). In the same line, Kraal et al. (2014) observed no differences in low- to moderate-risk patients entering cardiac rehabilitation in exercise functional capacity or quality of life during a 12-week home-based training program (61 ± 8 y) vs. a 12-week center-based training program (56 ± 9 y) (Kraal et al., 2014).

In addition, the study by Pratesi et al. (2019) compared a control group (standard cardiac rehabilitation for 4 weeks) versus a hybrid group (standard cardiac rehabilitation for 4 weeks + home-based exercise) in patients aged 75 years and older. The authors observed improvements in functional exercise capacity (VO2peak and, 6MWT distance walked) and lower limb muscle strength after 4 weeks of cardiac rehabilitation, similar benefits also observed in the present study. When evaluating the two groups at 6 and 12 months of follow-up, no differences were observed between the groups. This suggests that a home-based exercise program after face-to-face standard cardiac rehabilitation with monthly reinforcements does not add any long-term functional benefit beyond those offered by a conventional, 4-week outpatient cardiac rehabilitation program (Pratesi et al., 2019). Taken together, these studies show that supervised remote cardiac rehabilitation is effective in generating benefits for people with coronary artery disease.

In this study, we compared performing standard cardiac rehabilitation (12 weeks of face-to-face sessions) versus hybrid cardiac rehabilitation (6 weeks of face-to-face sessions + 6 weeks of remote monitoring). This decision is due to the fact that access to cardiac rehabilitation is limited in Chile (to a greater extent in public hospitals) due to limited human and physical resources. With such innovative proposals, we intend to be able to improve access for patients with coronary artery disease, especially older people, in the future. Also, it has been observed in cardiac surgery patients that hybrid cardiac rehabilitation is as effective as a hospital-based program in reducing pain components and it includes only 38% of the total cost in comparison to hospital-based delivery, so could be economically suitable to be also recommended to the coronary artery disease patients (Saeidi et al., 2017). In the same line, cardiac telerehabilitation intervention was likely to be cost-effective compared with center-based cardiac rehabilitation, suggesting that cardiac telerehabilitation maybe used as an alternative intervention for the treatment of patients with coronary artery disease (Brouwers et al., 2021).

Due to the associated comorbidities, medical referral to cardiac rehabilitation is less indicated in people over 60 years (Lutz and Forman, 2022). Age should not be a barrier since benefits of cardiac rehabilitation have been seen even in people over 80 years of age (Mehta et al., 2013). The risks will not outweigh the benefits of undergoing supervised cardiac rehabilitation.

Our study has limitations; the loss to follow-up due to the COVID-19 pandemic resulted in a high number of participants with missing measurements, which compromised the statistical power of knowing if there are differences between groups. For this reason, the findings should be taken with caution pending further studies.

## Conclusion

In conclusion, a hybrid exercise-based cardiac rehabilitation program could increase muscle strength and improve functional exercise capacity in adults and older people with coronary artery disease. More future studies comparing effectiveness among these age groups are needed to strengthen this conclusion.

## Data Availability

The original contributions presented in the study are included in the article/Supplementary Material, further inquiries can be directed to the corresponding author.
