# Supplementary material for: A hybrid exercise-based cardiac rehabilitation program is an effective strategy to improve muscle strength and functional exercise capacity in adults and older people with coronary artery disease
Source: Front Physiol. 2022 Aug 5;13:948273. doi: 10.3389/fphys.2022.948273 (PMC9389047; doi:10.3389/fphys.2022.948273)
Supplement: Supplementary file 1 [file Table1.DOCX]

# **Appendix 1**

# Hybrid Cardiac Rehabilitation Trial (HYCARET) project Office Staff, Centre coordinators, Investigators, and Key Staff.

**Project office (Universidad de La Frontera, Temuco, Chile)**

- Principal Investigator: P. Seron.
- First co-investigator: MJ Oliveros.
- International co-investigator: SL. Grace.
- Co-investigators: G. Marzuca-Nassr, F. Lanas, G. Morales, C. Román, S. Muñoz, N. Saavedra.
- Colaborators: D. Gomez, MJ Arancibia, C. Ulloa.

**Study sites, Chile:**

- **Antofagasta, Hospital de Antofagasta:** T. Marileo (center coordinador), I. Stavros, F. Muñoz, Y. Arias, O. Ferrada, C. González, H. Verdejo, MJ Segovia, K. Adaros, C. Aburto, F. Buzeta.
- **Santiago, Hospital Clínico Universidad de Chile:** R. Navarro (center coordinator) E. Valencia, JT Ramos, M. Osorio, F. Díaz.
- **Santiago, Hospital San Borja Arriarán:** G. Latín (center coordinator), S. Aravena, S. Gatta, JL Figueroa, D. Hermosilla.
- **Santiago, Hospital San José:** M. Gálvez (center coordinator), I. Díaz, N. Maldonado, R Navarro-Rañinao.
- **Santiago, Hospital San Juan de Dios:** JP Molina (center coordinator), V. Olea,
- **Temuco, Universidad de La Frontera:** P. Sepúlveda (center coordinator), R. Aravena, P. Sánchez, K. Morales. M. Saldias, F. Contreras.
